# New endoscopic ultrasonography criteria for malignant lymphadenopathy based on inter-rater agreement

**DOI:** 10.1371/journal.pone.0212427

**Published:** 2019-02-22

**Authors:** Yusuke Takasaki, Atsushi Irisawa, Goro Shibukawa, Ai Sato, Yoko Abe, Akane Yamabe, Noriyuki Arakawa, Takumi Maki, Yoshitsugu Yoshida, Ryo Igarashi, Shogo Yamamoto, Tsunehiko Ikeda

**Affiliations:** 1 Department of Gastroenterology, Aizu Medical Center, Fukushima Medical University, Aizuwakamatsu, Japan; 2 Department of Gastroenterology, Juntendo University, Tokyo, Japan; 3 Department of Gastroenterology, Dokkyo Medical University, Tochigi, Japan; 4 Department of Gastroenterology, Sendai Kousei Hospital, Sendai, Japan; Hospital Universitario de Canarias, SPAIN

## Abstract

**Background and aims:**

Various studies have been previously conducted on the diagnosis of lymphadenopathy as benign or malignant, but the results vary. These studies did not describe the inter-rater agreement on the EUS features of lymphadenopathy. In this study, we evaluate the inter-rater agreement on EUS features and propose EUS diagnostic norms for lymphadenopathy based on inter-rater agreement.

**Method:**

A total of 68 lymph nodes subjected to EUS-fine needle aspiration (FNA) were reviewed by five endoscopic experts. The EUS features evaluated lymph node size, shape, border, margin, echogenicity, homogeneity, and the hilum of the lymph node. Inter-rater agreement (multi-rater kappa statics) was performed. We established new criteria using results with a high degree of inter-rater agreement from EUS features and compared them with the former criteria.

**Result:**

There was a moderate agreement on shape, kappa (*K*) = 0.44 (95% confidence interval [CI]: 0.34–0.54), and fair agreement on echogenicity, homogeneity, border, and hilum of the lymph node, *K* (95% CI) = 0.33 (0.17–0.38), 0.34 (0.26–0.35), 0.22 (0.21–0.31), and 0.22 (0.11–0.26), respectively. This resulted in the establishment of new EUS diagnostic criteria using shape, long axis > 20 mm and short axis > 10 mm. New criteria were superior to old criteria (area under the curve 0.82 vs 0.52, P < 0.001).

**Conclusion:**

EUS diagnostic criteria for lymphadenopathy based on inter-rater agreement were more accurate than old criteria. This result will be useful for the diagnosis of lymphadenopathy.

## Introduction

Distinguishing between benign and malignant lymphadenopathies is important when determining a treatment plan. Endoscopic ultrasonography (EUS) is highly suited to assess lymphadenopathy in subjects with various gastrointestinal or thoracic malignancies [1–5]. Recently, EUS-guided fine-needle aspiration (EUS-FNA) has been remarkably developing and is actively employed for obtaining definitive diagnoses of lymphadenopathy observable via EUS from the gastrointestinal tract. EUS-FNA has a great ability of diagnosing lymphadenopathy, with recent reports describing a sensitivity and specificity of 74–92% and 100%, respectively [6,7]. In 2004, Chen et al.[8] described a high sensitivity and specificity (98.3% and 100%, respectively), and concluded that EUS-FNA is superior to lymph nodes (LN) echo features in evaluating lymphadenopathy. Nevertheless, EUS-FNA is a method for obtaining a pathological specimen that includes diagnostic imaging; thus, it is essential to first use imaging to infer whether the lesion is malignant or benign. Despite the diagnostic potential of EUS-FNA, it is not 100% precise, so a final diagnosis must be carried out not only on the basis of pathological findings but also by considering diagnostic imaging. Okasha et al.[6] indicated that additional complementary EUS features could be added to this technique for definitive diagnosis, although EUS-FNA is already a powerful tool in the diagnosis of benign and malignant LN. In addition, a greater understanding of the diagnostic imaging would facilitate ruling out unnecessary EUS-FNA, which can be considered important regarding safety and economy.

Catalano et al.[1] established that the EUS features which are predictive of malignancy in increasing order of importance were hypoechoic structure, sharply demarcated borders, rounded contour, and a size >10 mm. However, in similar studies, other results than those by Catalano et al. have been obtained. Hence, EUS diagnostic imaging of lymphadenopathy remains controversial because the EUS findings are not objective and are instead dependent on the subjective evaluation of the observer. In addition, EUS devices have lately become more advanced, with a large shift from the previous mechanical EUS to electronic EUS. The criteria using mechanical EUS may not match the diagnoses using electronic EUS. Thus, we performed a study employing a high degree of inter-rater agreement from the ultrasonographic findings adopted in the EUS diagnostic criteria for lymphadenopathy with the objective of proposing new diagnostic criteria for lymphadenopathy using EUS.

## Patients and methods

### 1. Study design

This was a retrospective evaluation of EUS diagnoses for lymphadenopathy in the mediastinal and abdominal cavities. The aim of this study was to establish new EUS diagnostic criteria for lymphadenopathy, benign or malignant. Considering the objective of this study, we first assessed the inter-rater agreement of the EUS features of lymphadenopathy. This study was reviewed and approved by the institutional review board of Fukushima Medical University and the institutional review board of Fukushima Medical University approved to waive the requirement for informed consent by opt out. This study was conducted in accordance with the human and ethical principles of research set forth in the Declaration of Helsinki.

### 2. Patients

Our subjects were 60 patients (with 68 lesions) who were subjected to a detailed EUS observation and EUS-FNA for lymphadenopathy observed in the mediastinal and abdominal cavities at Aizu Medical Center of Fukushima Medical University between June 2013 and August 2017. The final decision regarding benign or malign lymphadenopathy was made using a specimen collected employing EUS-FNA or a surgical resection sample. In particular, a benign determination in a non-resected case was made through an EUS-FNA established diagnosis of non-malignancy in addition to at least 6 months of follow-up observation.

### 3. Equipment

The echoendoscope and universal ultrasound processor used were the electronic scanner GF-UCP240, GF-UCT260, and EU-ME1/2 (Olympus Co., Tokyo, Japan) and the EG580UT and SU-1 (Fuji Film, Tokyo, Japan), respectively. The puncture needles used for EUS-FNA were EchoTip Ultra 25/22/19 gauge, ProCore 22 gauge (Cook Japan, Tokyo, Japan), SonoTip 22 gauge (Medicos Hirata, Tokyo, Japan), EZ Shot 2/3 22 gauge (Olympus Co.), Expect 22/25 gauge (Boston Scientific Japan, Tokyo, Japan), and Acquire 22 gauge (Boston Scientific Japan). The samples obtained using EUS-FNA were evaluated using histological and cytological diagnosis, but when sufficient core tissue was not sampled, the evaluation was performed using only a cytological diagnosis.

### 4. Evaluation of EUS features

For each patient, the still image recording clearly the punctured LN was selected (Y.T.) from the EUS images saved in the image filing system and applied in a blinded evaluation. The evaluation was performed by five endoscopic experts (board-certified fellows of the Japan Gastroenterological Endoscopy Society: A.I., G.S., A.S., Y.A., and A.Y.).

**Fig 1:**
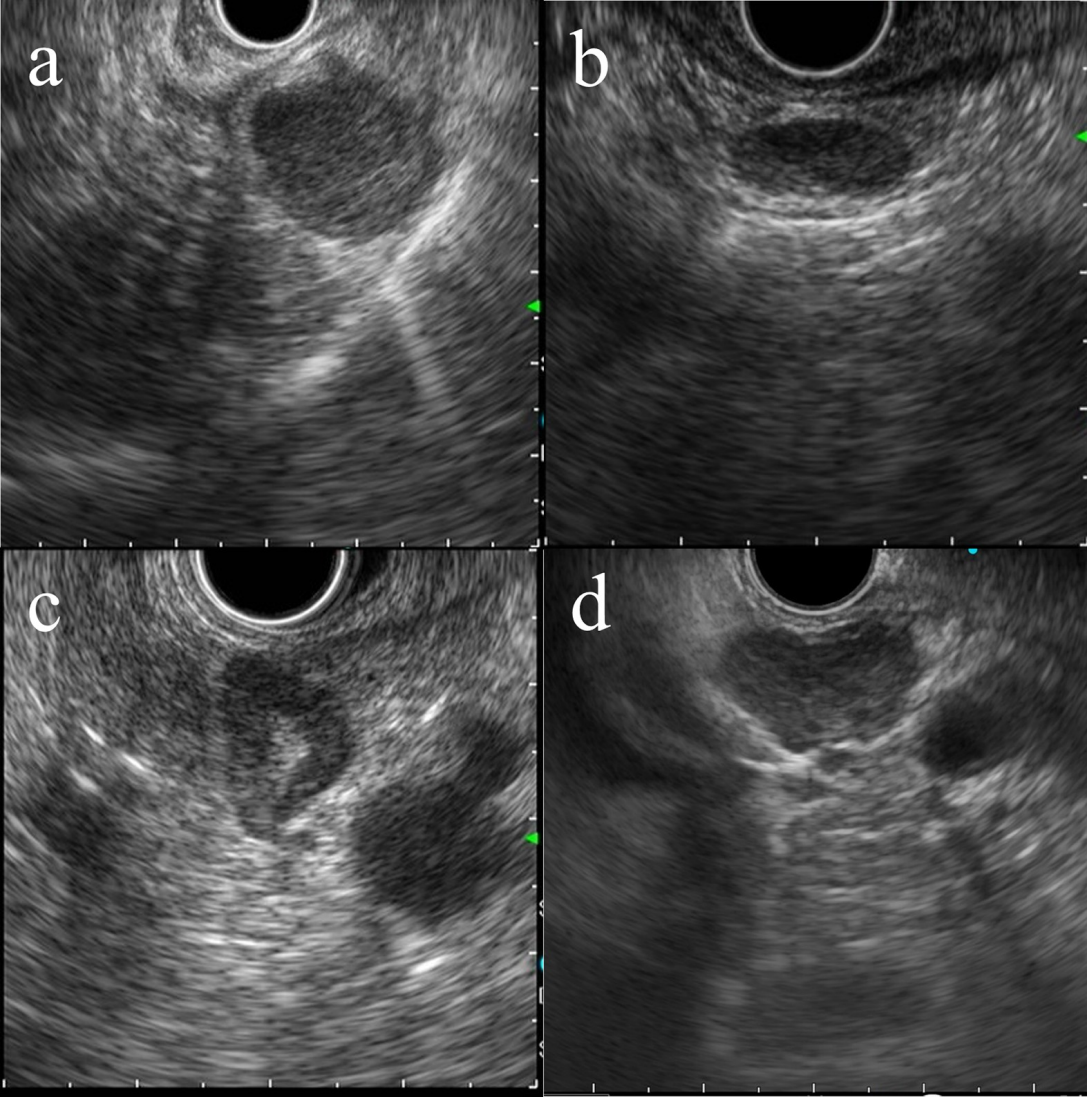
The shape classification of swollen lymph nodes on EUS image. (a) round shape, (b) oval shape, (c) triangle shape, and (d) polygonal shape.

**Fig 2:**
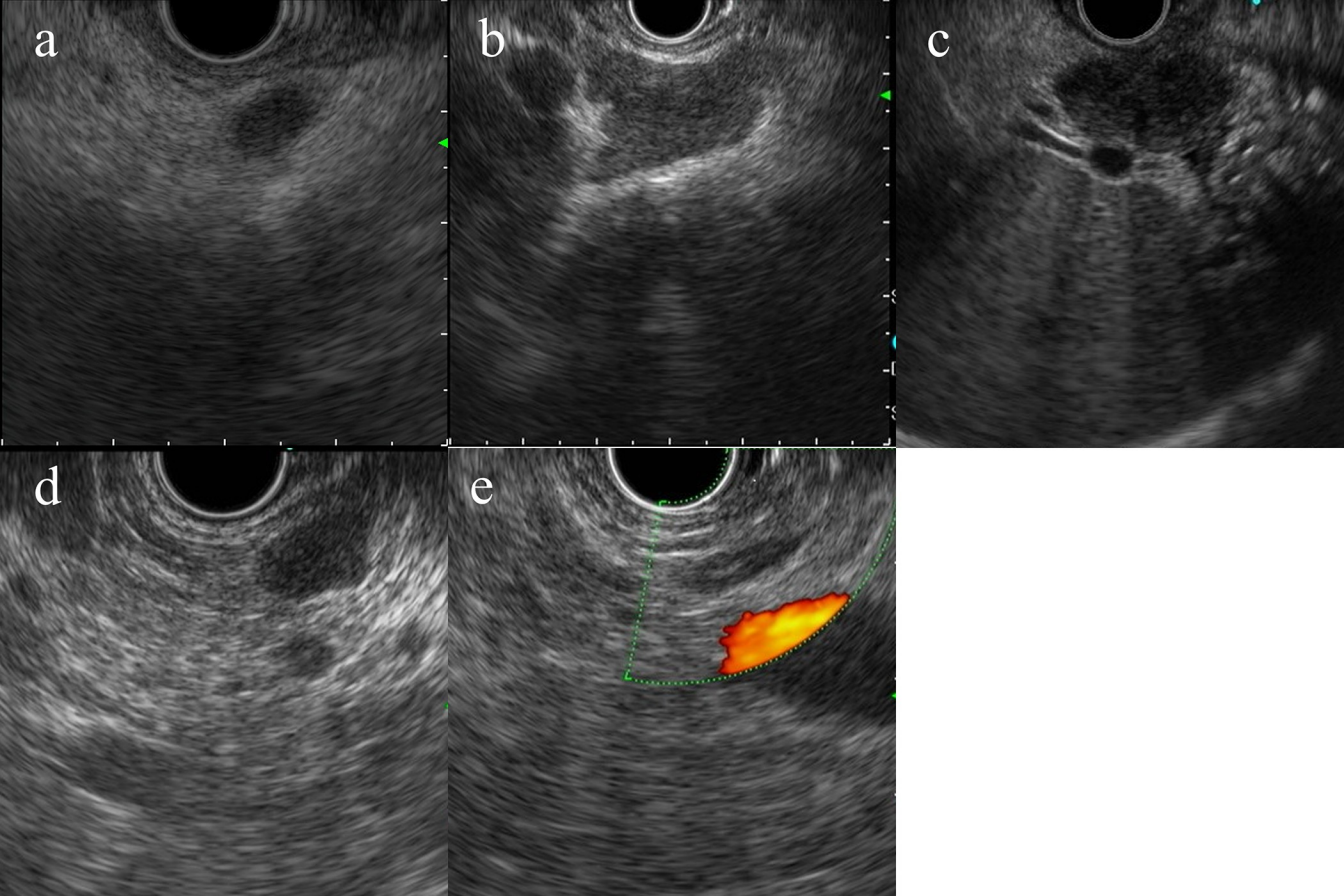
The definition of EUS feature of swollen lymph nodes. (a) fuzzy border, (b) irregular margin, (c) dark echogenicity, (d) homogeneous, (e) hilum of a lymph node.

The EUS features evaluated were the LN long-axis size, short-axis size, long-short ratio, shape, border, margin, echogenicity, homogeneity, and the presence of an internal hyperechoic component (hilum of the LN). The measurement of the long and short axes of the LN was performed by a separate individual who did not rate the images (N.A.). The shape was categorized as round, oval, triangular, or polygonal (Fig 1). The borders were classified as clear or fuzzy (Fig 2A); margins, as regular or irregular (Fig 2B); echogenicity, as dark or intermediate echoic (Fig 2C); and homogeneity, as homogenous or heterogeneous (Fig 2D). LN hilum was defined as having linear or band-shaped hyperechoic structures within lymphadenopathy (Fig 2E). Inter-rater agreement was calculated using multi-rater kappa statistics for these six EUS features. EUS image findings were agreed upon by three or more examiners. We evaluated the diagnostic ability of the resulting new EUS criteria in comparison with the previous criteria [1]. The previous criteria were a long axis > 10 mm, round shape, sharp border and hypoechoic pattern.

### 5. Statistical analysis

We marked the LN sizes as a mean ± SD. We calculated the categorical variables between the two groups by using the chi-square or Fisher test, and we compared continuous variables using the Wilcoxon rank-sum test. We performed a receiver-operating characteristic (ROC) analysis and determined the optimum cutoff values for the long and short axes of the LN. We used ROC analysis and the area under the curve (AUC) in comparison with the conventional criteria. In all the statistical analyses, the STATA Version 13 software (Stata Corp., College Station, TX, USA) was used.

## Results

### 1. Final diagnosis of lymphadenopathy

The mean age was 65.5 ± 13.7 years, the male-to-female ratio was 1.96:1, and the LN location was mediastinal in 8 cases and abdominal in 60 cases (Table 1).

**Table 1:** Comparison of EUS features between benign and malignant lymphadenopathy.

	Malignant LN (n = 30)	Benign LN (n = 38)	P value
Age, year	66.1±16.5	65.0±11.3	0.57
Male, %	22 (73.3)	23 (60.5)	0.27
Location (mediastinal), %	3 (10.0)	5 (13.2)	1.00*
Size (long axis), mm	25.3±11.3	16.8±8.1	**< 0.001**
Size (short axis), mm	16.3±9.6	9.8±5.2	**< 0.001**
Long-short ratio	0.66±0.21	0.59±0.17	0.15
Long-short ratio > 0.5, %	22 (73.3)	22 (57.9)	0.19
Long axis size > 10 mm, %	27 (90.0)	32 (84.2)	0.72*
Long axis size > 20 mm, %	19 (63.3)	9 (23.7)	**0.001**
Short axis size > 10 mm, %	24 (80.0)	12 (31.6)	**< 0.001**
Shape, %			
Round	8 (26.7)	4 (10.5)	0.11*
Oval	14 (46.7)	18 (47.4)	0.95
Triangle	0 (0.0)	5 (13.2)	0.06*
Polygonal	8 (26.7)	11 (29.0)	0.84
Round or oval (Long-short ratio > 0.5)	18 (60.0)	13 (34.2)	**0.03**

Note. Values presented with a plus/minus sign are means ± SD.

* Wilcoxon’s rank-sum test.

From the total number of subject lesions, 30 were instances of malignant lymphadenopathy (eight lesions of malignant lymphoma and 22 lesions of metastatic LN), while 38 were instances of benign lymphadenopathy. The typical breakdown of primary lesions for metastatic LN was five from gastric cancer, one from esophageal cancer, three from pancreatic cancer, and three from lung cancer (Table 2). Regarding location, three lesions of malignant lymphadenopathy were found in the mediastinum, while 27 were in the abdominal cavity. Meanwhile, five lesions of benign lymphadenopathy were in the mediastinum, while 33 were in the abdominal cavity.

**Table 2:** Primary lesion of malignant lymphadenopathy.

Primary lesion	
Gastric cancer	6 (20%)
Pancreatic cancer	3 (10%)
Lung cancer	3 (10%)
Ovary cancer	2 (7%)
Esophageal cancer	1 (3%)
Bile ductal cancer	1 (3%)
Neuroendocrine neoplasm (including MiNEN)	4 (13%)
Malignant lymphoma	7 (23%)
Unknown	3 (10%)

Note. MiNEN; mixed neuroendocrine-non-neuroendocrine neoplasm

### 2. Size of lymphadenopathy

The long axis was 25.3 ± 11.3 mm for malignant cases of lymphadenopathy and 16.8 ± 8.1 mm for benign cases. Thus, malignant lymphadenopathies were considerably larger (P < 0.001). The short axis in malignant cases was 16.3 ± 9.6 mm, while in benign cases it was 9.8 ± 5.2 mm. This indicates that malignant lymphadenopathies were significantly larger (P < 0.001; Table 1).

The analysis using ROC curves to determine the optimum cutoff value yielded values of 19.7 mm for the long axis and 10.7 mm for the short axis. Thus, when comparing benign and malignant cases using cutoff values of 20 mm for the long axis and 10 mm for the short axis, 19 (63.3%) of the malignant cases and 9 (23.7%) of the benign cases had a long axis of at least 20 mm (P = 0.001), while 24 (80.0%) of the malignant cases and 12 (31.6%) of the benign cases had a short axis of at least 10 mm (P < 0.001).

### 3. Significant EUS features based on inter-rater agreement

Based on the report by Landis et al.[9], we evaluated the inter-rater agreement for shape, border, margin, echogenicity, homogeneity, and hilum of the LN. Shape, with a kappa (*K*) value of 0.44 (95% confidence interval [CI]: 0.34–0.54), exhibited moderate inter-rater agreement. Echogenicity, homogeneity, border, and hilum of the LN had *K* values (95% CI) of 0.33 (0.17–0.38), 0.34 (0.26–0.35), 0.22 (0.21–0.31), and 0.22 (0.11–0.26), respectively, which represented fair inter-rater agreement. The margin had a K value (95% CI) of −0.02 (−0.07 to 0.02), which represented a low probability of agreement (Table 3).

**Table 3:** Inter-rater agreement of EUS features in 68 lymphadenopathies.

	Inter-rater agreement (Kappa)	95% CI	Assessment of inter-rater agreement
Shape	0.44	0.34–0.54	Moderate
Border	0.22	0.21–0.31	Fair
Margin	-0.02	-0.07–0.02	Less than chance
Echogenicity	0.33	0.17–0.38	Fair
Homogeneous	0.34	0.26–0.35	Fair
Hilum of lymph node	0.22	0.11–0.26	Fair

### 4. Making the new EUS criteria for lymphadenopathy

We analyzed the inter-rater agreement for six EUS features and size to produce new EUS criteria for lymphadenopathy. We analyzed the inter-rater agreement and used items with moderate inter-rater agreement (kappa [K] > 0.4) and size, which was found to have a statistically significant difference, to produce new EUS criteria for lymphadenopathy.

### 5. Evaluation of the new EUS criteria for lymphadenopathy

We evaluated the diagnostic ability of the resulting new EUS criteria in comparison with the previous criteria [1]. In doing so, we considered findings agreed upon by most evaluators to be the EUS findings for our subjects.

### 6. Proposal of EUS diagnostic criteria for lymphadenopathy

Based on the above-mentioned results, the creation of new EUS diagnostic criteria used the following three variables: “shape,” the item with moderate inter-rater agreement (*K* > 0.4), and lymphadenopathy size in terms of a long axis of at least 20 mm and a short axis of at least 10 mm, findings that exhibited a statistically significant difference. In accordance with previous reports [1,3,10–15], we used round or oval shapes with a long-to-short ratio of > 0.5 as shapes indicating malignant findings.

**Fig 3:**
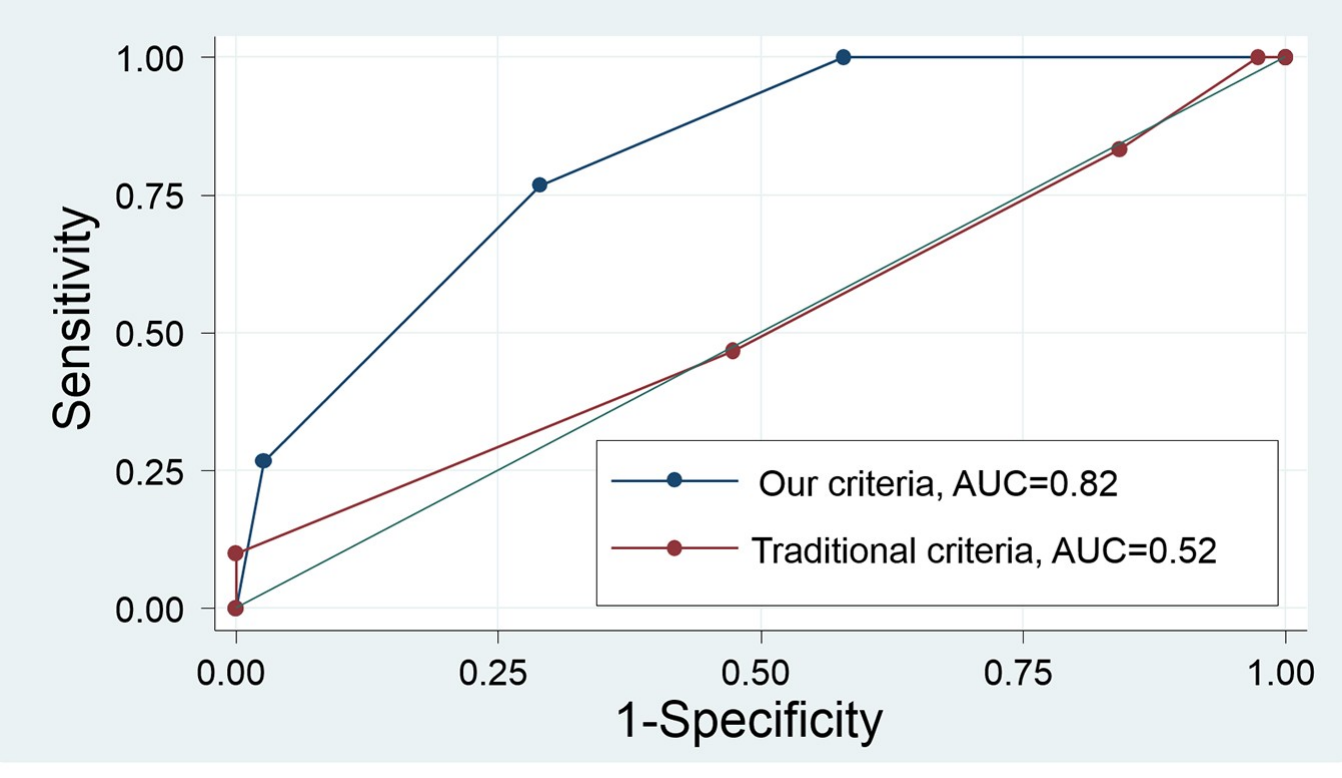
The ROC curve to compare our new proposed criteria (blue line) with the old criteria (red line). Our new proposed criteria were significantly superior to the old criteria (P < 0.001). Note. AUC; area under the curve.

We used the ROC curve to compare our new proposed criteria with the old criteria (long axis of at least 10 mm, round, hypoechoic, and clear border) in our subjects. Our new proposed criteria had an AUC of 0.82 (95% CI: 0.72–0.91), while the former criteria had an AUC of 0.52 (0.39–0.65). Thus, our new criteria were significantly superior (P < 0.001; Fig 3). In the analysis of the patients in this study, when at least two of our proposed criteria were present, the sensitivity was 76.7% and the specificity was 71.1% (positive predictive value 67.6% and negative predictive value 79.4%), while the positivity for all three criteria resulted in a sensitivity of 26.7% and a specificity of 97.4% (positive predictive value 88.9% and negative predictive value 62.7%). Meanwhile, the positivity for at least two of the old criteria resulted in a sensitivity of 83.3% and a specificity of 15.8% (positive predictive value 43.9% and negative predictive value 54.5%), while the positivity for at least three criteria resulted in a sensitivity of 46.7% and a specificity of 52.6% (positive predictive value 43.8% and negative predictive value 55.6%) (Table 4). In malignant lymphoma cases (n = 7), the validity of the criteria was maintained (S1 Table).

**Table 4:** Accuracy of the previously used criteria and our proposed criteria.

	sensitivity	specificity	Accuracy	PPV	NPV
at least two of the old criteria (95% CI)	83.3% (74.2–91.8)	15.8% (8.6–22.5)	45.6% (37.5–53.1)	43.9% (39.0–48.3)	54.5% (29.6–77.7)
at least three of the old criteria (95% CI)	46.7% (33.8–59.7)	52.6% (42.5–62.9)	50.0% (38.6–61.5)	43.8% (31.7–56.0)	55.6% (44.8–66.4)
at least two of our proposed criteria (95% CI)	76.7% (63.7–86.5)	71.1% (60.8–78.8)	73.5% (62.1–82.2)	67.6% (56.2–76.3)	79.4% (65.0–88.1)
all three of our proposed criteria (95% CI)	26.7% (17.7–29.4)	97.4% (90.3–99.5)	66.2% (58.2–68.6)	88.9% (58.9–98.0)	62.7% (58.1–64.1)

Note. PPV: Positive predictive value, NPV: Negative predictive value, CI: confidence interval

## Discussion

Various studies have been already conducted on the diagnosis of lymphadenopathy as benign or malignant. Initially, this was mainly an imaging-based diagnosis. However, in the recent years, EUS-FNA has undergone an impressive development, which plays a major role in examining lymphadenopathy that can be observed from the gastrointestinal tract. EUS-FNA has a high diagnostic ability for lymphadenopathy, and recent reports have cited a sensitivity and specificity of 74–92% and 100%, respectively[6,8]. Even in 2004, Chen et al.[8] demonstrated high sensitivity and specificity (98.3% and 100%, respectively), and concluded that EUS-FNA is superior to LN echo features in evaluating lymphadenopathy. However, EUS-FNA is a pathological sample collection method that includes diagnostic imaging, so it is necessary to first infer whether the lesion is malignant or benign by using imaging. The diagnostic ability of EUS-FNA is high, but not of 100%, so the final diagnosis must be made by considering the imaging results without a slavish adherence to the pathological diagnosis. Okasha et al.[6] indicated that additional complementary EUS features could be added to this technique for a definitive diagnosis, although EUS-guided FNA cytology is a powerful modality in the diagnosis of benign and malignant LN. In addition, an understanding of the diagnostic imaging would lead to an avoidance of unnecessary EUS-FNA, which can be considered quite significant in terms of safety and economy. Moreover, the high specificity of the new criteria can be of great help when taking the decision to do a re-biopsy of lesions with a negative FNA result while having positive signs of malignancy rather than avoiding unnecessary biopsies.

Our study is the first to propose EUS diagnostic criteria for lymphadenopathy based on inter-rater agreement. EUS is an extremely useful diagnostic tool capable of high-resolution observations of subjects at a close range through the gastrointestinal tract. However, EUS findings are dependent on the subjective interpretation of the observer; thus, difficulty in making evaluations with sufficient objectivity has been an issue. EUS is also widely implemented as a minimally invasive test for pancreatic disorders. However, in that context, inter-observer/inter-rater agreement was an issue, which led to studies seeking objectivity[16–18]. As for the inter-rater agreement in EUS diagnostic imaging for lymphadenopathy, de Melo et al.[10] studied the agreement between three experts with > 5000 cases of EUS experience, reporting a fair agreement on shape, with a *Κ* value of 0.35 (95% CI: 0.2–0.5), and moderate agreement on echogenicity and borders, with a *Κ* value of 0.46 (95% CI: 0.31–0.61) and 0.43 (95% CI: 0.27–0.58), respectively. Meanwhile, our research found moderate agreement on shape, with a *K* value of 0.44 (95% CI: 0.34–0.54), while echogenicity, with a *K* value of 0.33 (95% CI: 0.17–0.38), and border, with a *K* value of 0.22 (95% CI: 0.21–0.31), had a low probability of agreement. Even from these two studies, the EUS diagnostic imaging of lymphadenopathy is clearly ambiguous.

In the previously reported diagnoses of lymphadenopathy using EUS, all the reports construct criteria by citing significant findings for malignancy, but they do not discuss inter-rater agreement, which may affect differences in results between papers. Indeed, we investigated the inter-rater agreement of a clear border, hypoechoic, and round shape as EUS imaging features of malignant lymphadenopathy that were reported by Catalano et al.[1] and used by numerous studies. Owing to our finding that these had fair agreement aside from shape, these criteria, which have been used by various endoscopists, can be considered problematic. In our study, we constructed simple criteria by combining the long- and short-axis size and shape of the LN as the finding with at least a moderate agreement among the raters, who were board-certified fellows of the Japan Gastroenterological Endoscopy Society with a thorough knowledge regarding EUS diagnostic imaging. Although these criteria were somewhat inferior in sensitivity and specificity to those mentioned earlier for EUS-FNA, nonetheless they exhibited a higher sensitivity and specificity than the previously reported diagnostic imaging techniques. Moreover, reports on the “size” variable include that by Chen et al.[7], who compared EUS-FNA and EUS findings for 183 LN and reported that the short axis was a significant indicator for malignancy. Unlike the EUS features, including shape, border, margin, echogenicity, and homogeneity, size is an objective factor, so its addition to the diagnostic criteria is important.

Additionally, we referred to previous reports on what findings indicated malignancy for the shape variable, which we used in our study. As stated earlier, many reports have suggested a correlation between a round shape and malignant cases[1,3,10–12]. Hocke et al.[13] treated a polygonal shape as an indicator of a benign LN. Bhutani et al. [14] only found one case of a triangular shape among 16 malignant cases, suggesting that the shape indicates benignity. Indeed, the malignant cases in our study included no triangle shapes. In addition, Tohnosu et al.[15] reported that a LN long-to-short ratio of > 0.5 significantly correlated with a malignant lymphadenopathy. We defined malignant findings for shape as round or oval with a long-to-short ratio of > 0.5 based on these reports.

The limitations of our study are as follows: First, it was a single-center retrospective study with a small sample size. Thus, our results need to be validated with other data sets of large cases in a prospective study. Second, the transducer processors used for EUS are not uniform. In particular, if the same EUS observation device was to be used, a high inter-rater agreement would be obtained for internal echogenicity and homogeneity, which had been previously considered being significant imaging findings. Third, the evaluation of EUS imaging was performed using only one clear picture. EUS imaging was a dynamic evaluation, so if we evaluated EUS imaging using a movie or in real-time, a high inter-rater agreement would have been obtained for border and shape. In the future, a prospective study eliminating these limitations will be needed.

The present study once again highlighted the weaknesses of EUS diagnostic imaging. Indeed, even if individual EUS findings can be defined in writing, the main problem that EUS diagnostic imaging involves a morphological evaluation remains, and so does the subjective judgment of the observer. Our study combined size and the finding “shape,” for which moderate agreement was obtained, to propose criteria with a reasonable diagnostic ability as a form of diagnostic imaging. However, EUS imaging includes various factors that reflect real pathological findings. With that in mind, one would expect that more-accurate criteria could be constructed by including more variables. To achieve this, an objective evaluation of each variable using a digital image analysis would be necessary. Recently, many studies have been conducted along these lines[19,20]. Zhu et al.[21] reported that digital image processing and computer-aided EUS image differentiation technologies are highly accurate and noninvasive in differential diagnosis between pancreatic cancer and chronic pancreatitis. Further advances in such techniques could give rise to rapid improvements in the accuracy of EUS diagnostic imaging, which currently has a tendency toward ambiguity. Our present study suggests that support for diagnostic imaging using digital image analysis could be a major theme for future research.

## Supporting information

S1 Table Accuracy of old and our proposal criteria in malignant lymphoma patients. (DOCX)

S1 Dataset (DOCX)
