# Supplementary material for: New endoscopic ultrasonography criteria for malignant lymphadenopathy based on inter-rater agreement
Source: PLoS One. 2019 Feb 22;14(2):e0212427. doi: 10.1371/journal.pone.0212427 (PMC6386303; doi:10.1371/journal.pone.0212427)
Supplement: S1 Table — (DOCX) [file pone.0212427.s001.docx]

|  | sensitivity | specificity | Accuracy |
| --- | --- | --- | --- |
| at least two of the old criteria | 83.3% | 15.8% | 45.6% |
| at least three of the old criteria | 46.7% | 52.6% | 50.0% |
| at least two of our proposed criteria | 76.7% | 71.1% | 73.5% |
| all three of our proposed criteria | 26.7% | 97.4% | 66.2% |

**S1 Table.** **Accuracy of old and our proposal criteria in malignant lymphoma patients**
