# Supplementary material for: New endoscopic ultrasonography criteria for malignant lymphadenopathy based on inter-rater agreement
Source: PLoS One. 2019 Feb 22;14(2):e0212427. doi: 10.1371/journal.pone.0212427 (PMC6386303; doi:10.1371/journal.pone.0212427)
Supplement: S1 Dataset — (DOCX) [file pone.0212427.s002.docx]

Data file

https://doi.org/10.6084/m9.figshare.7699349, <https://doi.org/10.6084/m9.figshare.7699355>

https://doi.org/10.6084/m9.figshare.7550171
